# Lipoprotein *N*-Acylation in *Staphylococcus aureus* Is Catalyzed by a Two-Component Acyl Transferase System

**DOI:** 10.1128/mBio.01619-20

**Published:** 2020-07-28

**Authors:** John H. Gardiner, Gloria Komazin, Miki Matsuo, Kaitlin Cole, Friedrich Götz, Timothy C. Meredith

**Affiliations:** a The Huck Institutes of the Life Sciences, The Pennsylvania State University, University Park, Pennsylvania, USA; b Department of Biochemistry and Molecular Biology, The Pennsylvania State University, University Park, Pennsylvania, USA; c Microbial Genetics, Interfaculty Institute of Microbiology and Infection Medicine Tübingen, University of Tübingen, Tübingen, Germany; Emory University School of Medicine; University of Georgia

**Keywords:** *Staphylococcus*, *Staphylococcus aureus*, TLR2, acyl transferases, immune response, lipoproteins, Toll-like receptors

## Abstract

Bacterial lipoproteins (Lpps) are a class of membrane-associated proteins universally distributed among all bacteria. A characteristic N-terminal cysteine residue that is variably acylated anchors C-terminal globular domains to the extracellular surface, where they serve numerous roles, including in the capture and transport of essential nutrients. Lpps are also ligands for the Toll-like receptor 2 (TLR2) family, a key component of the innate immune system tasked with bacterial recognition. While Lpp function is conserved in all prokaryotes, structural heterogeneity in the N-terminal acylation state is widespread among *Firmicutes* and can differ between otherwise closely related species. In this study, we identify a novel two-gene system that directs the synthesis of *N*-acylated Lpps in the commensal and opportunistic pathogen subset of staphylococci. The two genes, which we have named the lipoprotein *N*-acylation transferase system (Lns), bear no resemblance to previously characterized N-terminal Lpp tailoring enzymes. LnsA (SAOUHSC_00822) is an NlpC/P60 superfamily enzyme, whereas LnsB (SAOHSC_02761) has remote homology to the CAAX protease and bacteriocin-processing enzyme (CPBP) family. Both LnsA and LnsB are together necessary and alone sufficient for *N*-acylation in Staphylococcus aureus and convert the Lpp chemotype from diacyl to triacyl when heterologously expressed in Listeria monocytogenes. Acquisition of *lnsAB* decreases TLR2-mediated detection of S. aureus by nearly 10-fold and shifts the activated TLR2 complex from TLR2/6 to TLR2/1. LnsAB thus has a dual role in attenuating TLR2 signaling in addition to a broader role in bacterial cell envelope physiology.

## Abstract (precis)

Although it has long been known that S. aureus forms triacylated Lpps, a lack of homologs to known *N*-acylation genes found in Gram-negative bacteria has until now precluded identification of the genes responsible for this Lpp modification. Here, we demonstrate N-terminal Lpp acylation and chemotype conversion to the tri-acylated state is directed by a unique acyl transferase system encoded by two noncontiguous staphylococci genes (*lnsAB*). Since triacylated Lpps stimulate TLR2 more weakly than their diacylated counterparts, Lpp *N*-acylation is an important TLR2 immunoevasion factor for determining tolerance or nontolerance in niches such as in the skin microbiota. The discovery of the LnsAB system expands the known diversity of Lpp biosynthesis pathways and acyl transfer biochemistry in bacteria, advances our understanding of Lpp structural heterogeneity, and helps differentiate commensal and noncommensal microbiota.

## INTRODUCTION

Bacterial lipoproteins (Lpps) are ubiquitous components of bacterial cell membranes containing a characteristic lipid-modified N-terminal cysteine residue that anchors C-terminal globular protein domains to the cell surface (1–3). Lpps comprise, on average, ∼2.7% of all prokaryotic open reading frames and perform a wide variety of cellular functions occurring at the interface of the extracellular bacterial surface with the environment (4). In the Gram-positive opportunistic pathogen Staphylococcus aureus, there are approximately 70 total Lpp-encoding genes (5, 6), of which a highly conserved 60-member subset is present across genetically diverse lineages. While cellular roles for much of the lipoproteome remain uncharacterized, many Lpps are involved in the uptake of essential nutrients, as well as in maintaining general bacterial fitness and virulence. Iron capture and transport is a particularly critical factor in establishing infections, and S. aureus has devoted multiple Lpps for this purpose (7, 8). The accessory *lpp* gene subset is more limited, being scattered throughout the core genome and within pathogenicity-associated islands in certain virulent S. aureus isolates. Tandem-type Lpps (also called lipoprotein-like [Lpl] or DUF576 proteins), for instance, are an abundant paralogous gene family present in multicopy clusters in discrete loci throughout the genome (5). It has been suggested that Lpl diversity is the result of recombination-driven antigenic variation (9) and that Lpl promotes host cell invasion by binding to Hsp90 (10).

The integral roles of Lpps in bacterial physiology and fitness, in combination with their abundance, extracellular locale, conserved structure, and widespread distribution among all bacteria, make them a key focal point for recognition by the innate immune system (11). A unique thioether-linked diacylglycerol moiety attached to the N-terminal cysteine of Lpp is recognized by heterodimeric Toll-like receptor 2 (TLR2) complexes, a group of integral membrane proteins expressed by macrophages and other immune-related cells (12). The TLR2 proteins (TLR2/TLR1/TLR6) are pattern recognition receptors with leucine-rich repeat ectodomains that bind external Lpp ligand, leading to the dimerization of intracellular Toll–interleukin-1 receptor (TIR) homology domains. TIR domain dimerization activates the MyD88-dependent intracellular signaling pathway, inducing translocation of nuclear factor-κB (NF-κB) and proinflammatory cytokine secretion to help clear the bacterial infection (13–15). A thioether-linked acylated glycerol substituent is common to all bacterial Lpp chemotypes and is the core ligand recognition feature of TLR2. The state of the N-terminal cysteine dictates the TLR2 dimerization partner (TLR1 or TLR6). In most Gram-negative bacteria, the N terminus is modified to form triacylated Lpp (TA-Lpp) (2). The amide-linked acyl chain is critical for substrate recognition and efficient trafficking to the outer membrane by the Lpp transport system (Lol) (16). In the TLR2/1 heterodimer, TLR2 binds the diacylglycerol region, while the hydrophobic pocket in TLR1 accommodates the *N*-acyl chain (17). Many Gram-positive bacteria, which lack an outer membrane and do not need Lol-mediated transport, retain diacylated Lpps with free α-amino termini (DA-Lpps). DA-Lpps are recognized by TLR2/TLR6 heterodimers, with TLR2 binding diacylglycerol and TLR6 interacting with the α-amino group (18).

While it was previously thought that DA-Lpp from Gram-positive and TA-Lpp from Gram-negative bacteria represented all bacterial Lpp chemotypes, novel structures have since been discovered. In a survey of the Gram-positive phylum *Firmicutes*, *N-*acetyl, *N-*peptidyl, lyso-Lpp (lyso-form lipoprotein), and *N-*acylated TA-Lpp similar in structure to Gram-negative bacteria have now been characterized (19). Clearly, N-terminal modification has a purpose beyond Lpp trafficking to the outer membrane. We recently identified the lipoprotein intramolecular transferase (Lit) enzyme in Enterococcus faecalis that catalyzes the internal migration of the *sn-*2 acyl chain of the di-acylglycerol moiety in DA-Lpp substrate to the *N* terminus (20). The resulting lyso-Lpp chemotype still retains two total acyl chains but becomes a much weaker TLR2/6 ligand, and almost undetectable by TLR2/1 activity despite being *N*-acylated (21). The TLR2/1/6 response to lyso-Lpp decreases 100- to 1,000-fold when challenged with either model lipopeptides or heat-killed whole cells as ligands. In comparison to the DA-Lpp-producing type strain of Listeria monocytogenes, much higher relative bacterial cell concentrations of E. faecalis are needed to trigger a TLR2 response (21). Lpp chemotype conversion within a species can also attenuate TLR2 activity. Certain L. monocytogenes environmental isolates have acquired a plasmid-borne, copper-inducible *lit2* paralog that induces a weaker TLR2 response when grown in copper-supplemented media (21). Among staphylococci, Staphylococcus carnosus forms *N*-acetylated Lpps and induces 10-fold higher levels of the proinflammatory IL-6 cytokine than does S. aureus with TA-Lpp (22, 23). Differences in immunostimulation among Lpp chemotypes thus helps to define the potential for virulence, as well as to facilitate the niche-specific adaptation of commensal bacteria from closely related noncommensal isolates (23).

Many of the enzymes directing Lpp N-terminal tailoring reactions, which in turn can modulate host TLR2 responses, remain to be discovered. Of particular note, S. aureus synthesizes TA-Lpp despite lacking an apparent ortholog to the *N*-acyl transferase (Lnt) used in Gram-negative bacteria (24–26). Schneewind and coworkers first reported on a base-stable Lpp acyl group, suggesting the presence of an amide-linked *N*-acyl chain (27). Analysis by mass spectrometry subsequently followed and provided direct structural evidence that unequivocally confirmed the TA-Lpp chemotype in S. aureus (28, 29). Here, we report the identification of two previously uncharacterized genes (SAOUHSC_00822 and SAOUHSC_02761) in S. aureus required for this Lpp *N*-acylation using a TLR2/1-specific reporter assay to screen a random transposon library. We named this novel two-component Lpp tailoring machinery the Lipoprotein *N*-acyl transferase system (LnsA and LnsB). Neither LnsA or LnsB share any sequence similarity to Lnt or Lit, and both are only present in *Staphylococcus* species thus far known to make TA-Lpp. We show that loss of either gene in S. aureus converts TA-Lpp to DA-Lpp and that both genes are absolutely required and together sufficient to convert DA-Lpp to TA-Lpp in the L. monocytogenes. In either host, TLR2 challenge with DA-Lpp-producing strains induced a more potent response relative to isogenic TA-Lpp counterparts. Deletion of either *lnsA* or *lnsB* increased interleukin-8 (IL-8) secretion nearly 10-fold, indicating LnsAB are important determinants acquired by commensal and opportunistic staphylococci pathogens to evade TLR2 immune surveillance. The discovery of the presumptive two-component LnsAB complex expands the known diversity of Lpp tailoring reactions in bacteria.

## RESULTS

### Transposon library screen for diminished TLR2/1 signaling.

We initially hypothesized that a single integral membrane protein in Staphylococcus aureus performs an analogous function to Lnt from Escherichia coli. Our selection strategy using growth rescue of an E. coli Lnt-depletion strain, however, that had successfully identified *lit* from Enterococcus faecalis and Bacillus cereus genomic DNA libraries (20) repeatedly failed when using S. aureus genomic DNA as the input library (data not shown). We thus turned to an indirect phenotypic screen to monitor loss of TLR2/1 signal, which has significantly higher affinity for TA-Lpp than DA-Lpp ligand, paired with a TLR2/6 specific activity counterscreen to eliminate candidates expressing either less total Lpp or that grew to a lower final biomass. Colonies from a high-coverage S. aureus mariner transposon (Tn) library built in the model lab strain NCTC8325 were used in the TLR2 reporter assays (30). We screened ∼4,000 Tn mutants, and identified two unique Tn insertions in proximity to the unidentified open reading frame SAOUHSC_02761 (see Fig. S1 in the supplemental material). SAOUHSC_02761 is predicted to be a polytopic integral membrane protein by TMHMM2.0 (31), and has no sequence similarity with functionally annotated conserved domains. Remote protein homology with CAAX protein proteases (32) can be detected using HHPred, an algorithm that considers structure, as well as sequencing, to identify distant homology (33). The first Tn mutant (Tn 16C2) inserted 18 bp upstream from the SAOUHSC_02761 start codon, while the second (Tn 32F1) disrupted the coding region (amino acid 114 of a 249-amino-acid SAOUHSC_02761 ORF) (Fig. S1A). Northern blotting using an antisense SAOUHSC_02716 N-terminal probe confirmed a monocistronic transcript, that Tn 16C2 prevents readthrough expression, and that 32F1 expresses a longer transcript that is predicted to be frameshifted (Fig. S1B). Both Tn mutants had markedly diminished TLR2/1-specific stimulating activity comparable to disruption of the other known Lpp biosynthetic pathway enzymes, lipoprotein diacylglycerol transferase (Lgt) and lipoprotein signal peptidase II (Lsp) (Fig. S1C). Lgt links diacylglycerol through a thioether bond using a neighboring phospholipid to make preapolipoprotein on the extracellular membrane surface (34, 35), which Lsp then cleaves to liberate the free α-amino cysteine terminus and complete DA-Lpp formation (36). Unlike Tn insertion in *lgt* and *lsp*, both Tn 16C2 and 32F1 mutants retained TLR2/6 activity at least as active as wild type (Fig. S1D). This is consistent with a common loss of SAOUHSC_02716 function genotype for both Tn mutants.

10.1128/mBio.01619-20.2 FIG S1 Transposon insertions in S. aureus NCTC8325 strain TM226 that attenuate TLR2 activity. (A) Both Tn insertions from the TM226 library conferring reduced TLR2/1 activity inserted in the locus containing the unknown open reading frame SAOUHSC_02761. Insertion sites were mapped by inverse PCR of *Taq*αI-digested genomic DNA that had been recircularized by ligation. Tn 16C2 inserted 18 bp upstream from the start codon of SAOUHSC_02761 and Tn 32F1 inserted within amino acid 114 of the 249-amino-acid open reading frame. The SAOUHSC_00822 locus is also shown for reference. Location of putative promoters (*P*) and transcriptional terminators (TT) are annotated based on data from AureoWiki (S. Fuchs, H. Mehlan, J. Bernhardt, A. Hennig, S. Michalik, K. Surmann, J. Pane-Farre, A. Giese, S. Weiss, L. Backert, A. Herbig, K. Nieselt, M. Hecker, U. Volker, and U. Mader, Int J Med Microbiol 308:558–568, 2018, https://doi.org/10.1016/j.ijmm.2017.11.011). (B) Northern blot analysis of total RNA extracted from wild-type TM226 (Wt) and the Tn insertion mutants (Tn 16C2 and Tn 32F1). A biotin-labeled antisense transcript annealing to the *N terminus* of SAOUHSC_02761 (blue bar in panel A) was used to detect mRNA. A second blot was performed using antisense probe directed to the interior of the SAOUHSC_00822 open reading frame did not reveal any changes in expression with Tn insertions in SAOUHSC_02761. Approximate size of transcripts is indicated. (C) The TLR2/1-specific activity was measured using heat-killed extracts from the wild-type TM226 (Wt) and Tn insertion mutants in either *lsp* or *lgt*. Both Tn insertion mutants (Tn 16C2 and Tn 32F1) weakly induce TLR2/1. Control lipoprotein chemotypes (tri-acylated [TA-Lpp] and di-acylated [DA-Lpp]) were prepared from E. coli donor strain extracts. Error bars represent the standard deviation results of at least three replicates. (D) The TLR2/6 specific activity was measured as in panel C. (E) PCR amplification of all loci using primers (∼1 kb up- and downstream of the coding region) flanking either *lgt*, *lsp*, SAOUHSC_02761, or SAOUHSC_00822 confirmed the anticipated genotypes. *, Misamplification product. Download FIG S1, TIF file, 0.6 MB. Copyright © 2020 Gardiner et al. 2020 Gardiner et al. This content is distributed under the terms of the Creative Commons Attribution 4.0 International license.

**FIG 1:**
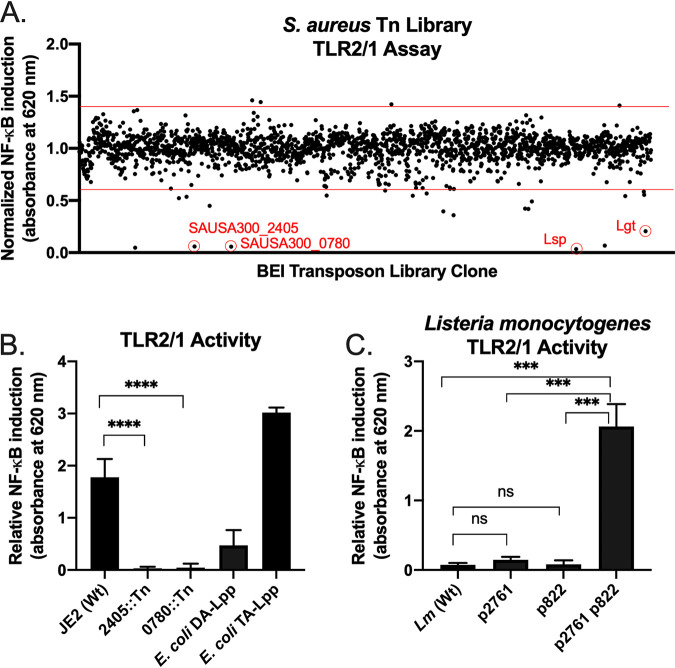
TLR2 activity of S. aureus USA300 Tn mutants. (A) Heat-killed extracts were prepared from all 1920 Tn mutants in the NTML prearrayed library. Induction of NF-κB was measured colorimetrically through secretion of alkaline phosphatase. Raw absorbance values were normalized to the average absorbance across the entire library. Among the Tn mutants demonstrating changes in absorbance exceeding the norm by more than three standard deviations (red horizontal lines), four hits were robust and could be consistently replicated. Two were in known enzymes involved in Lpp biosynthesis (*lgt*::Tn and *lsp*::Tn), while two were in uncharacterized open reading frames [NE536 (SAUSA300_0780::Tn) and NE407 (SAUSA300_2405::Tn)]. (B) The TLR2/1 specific activity was measured for both new genetic determinants (SAUSA300_0780::Tn and SAUSA300_2405::Tn) and compared to wild type (JE2) and Lpp control chemotypes (triacylated [TA-Lpp] and diacylated [DA-Lpp]) from E. coli donor strains. Error bars represent the standard deviation results of at least three replicates. (C) The TLR2/1 specific activity was measured from heat-killed extracts from wild-type L. monocytogenes (expressing DA-Lpp) and isogenic strains expressing SAOUHSC_02761 (same as SAUSA300_2405, p2761), SAOUHSC_00822 (same as SAUSA300_0780, p822), or both genes together (p2761, p822). Statistical significances were calculated by using Student *t* tests (*, *P* < 0.05; **, *P* < 0.01; ***, *P* < 0.001; ****, *P* < 0.0001; ns, not significant).

Although the immunoassay data implicated SAOUHSC_02761 in TA-Lpp formation, introducing SAOUHSC_02761 into an E. coli Lnt depletion strain failed to rescue growth (data not shown). We presumed an issue with heterologous expression or the available fatty acid donor pool in E. coli, so we repeated the TLR2 immunoassay using L. monocytogenes. Both S. aureus and L. monocytogenes have saturated branched-chain fatty acids (37, 38), utilize acyl-phosphate donors in glycerophospholipid biosynthesis (39, 40), and in general share much cell envelope physiology. However, introduction of SAOUHSC_02761 once again failed to induce phenotypic conversion (see below), indicating SAOUHSC_02761 may be required for TA-Lpp formation but not alone sufficient. We took advantage of the prearrayed Nebraska Transposon Mutant Library (NTML) in the S. aureus JE2 USA300 clinical isolate to repeat the TLR2/1 specific immunoactivity screen (Fig. 1A). We identified six Tn gene disruption mutants with decreased activity, including in SAUSA300_2405 [Tn NE407(Tn*2405*) or SAOUHSC_02761 in strain NCTC8325], *lgt*, and *lsp*. The only Tn library mutant besides SAUSA300_2405 that retained TLR2/6 activity while growing to a normal final biomass had an insertion in the uncharacterized open reading frame SAUSA300_0780 [Tn NE536(Tn*0780*) or SAOUHSC_00822 in strain NCTC8325]. The SAUSA300_0780/SAOUHSC_00822 gene encodes a 189-amino-acid protein containing a domain with very weak similarity to the NlpC/P60 endopeptidase superfamily (41) and is predicted by SignalP v5.0 to contain a signal peptide for extracellular transport (42). As with SAUSA300_2405/SAOUHSC_02761, this gene is expressed under standard culture conditions but is part of a polycistronic operon as judged by transcript length (Fig. S1B). Expression levels of SAUSA300_0780/SAOUHSC_00822 were constant and not reliant on SAUSA300_2405/SAOUHSC_02761 function. Tn insertion in either SAUSA300_0780 or SAUSA300_2405 in S. aureus USA300 JE2 decreased TLR2/1 activation by >50-fold compared to the wild type (Fig. 1B).

To confirm we had identified all candidate genes required for synthesis of a TLR2/1-active Lpp chemotype, we heterologously expressed both SAOUHSC_00822 and SAOUHSC_0276 in L. monocytogenes. L. monocytogenes normally makes DA-Lpp, so introduction of both candidate genes from S. aureus should impart TLR2/1 activity. While either gene alone did not produce TLR2/1 signal, the expression of both genes with a constitutive promoter markedly enhanced the TLR2/1 response (Fig. 1C). Although other genes (such as those providing the acyl donor) may be required for Lpp chemotype conversion, these genes are evidently not specific to S. aureus.

### Deletion of either SAOUHSC_00822 or SAOUHSC_02761 decreases detection by TLR2/1 while enhancing TLR2/6 activity.

**FIG 2:**
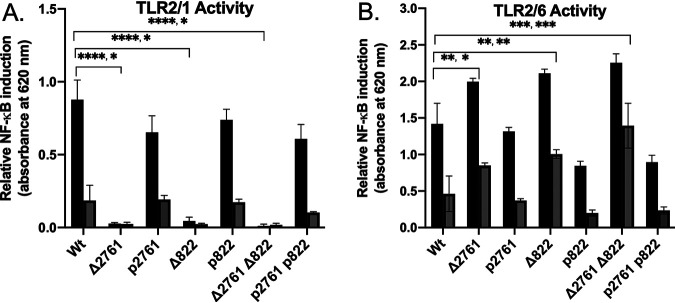
TLR2 activity of gene deletion mutants in S. aureus NCTC8325. TLR2 activation through either TLR2/1 or TLR2/6 was measured by NF-κB induction. (A) TLR2/1 receptor activity was measured in parent strain S. aureus TM226 (Wt), in single gene deletion mutants (ΔSAOUHSC_02761 [Δ2761], Δ2761 with plasmid back-complementation [p2761], ΔSAOUHSC_00822 [Δ822], and Δ822 with plasmid back-complementation [p822]), and in double gene deletion mutants (Δ2761Δ822, Δ2761Δ822 with plasmid back-complementation [p2761, p822]). Heat-killed bacterial extracts were applied either as concentrated (black) or 5-fold diluted (gray) aliquots. (B) TLR2/6 receptor activity using the same extracts as in panel A, except that concentrated (black) or 10-fold-diluted (gray) aliquots were used. Error bars in both panels represent standard deviation results of at least three experimental replicates. Statistical significances (listed for black and gray bars) were calculated by using Student *t* tests (*, *P* < 0.05; **, *P* < 0.01; ***, *P* < 0.001; ****, *P* < 0.0001).

To determine whether the decrease in TLR2/1 signal was solely due to Tn disruption and whether the two mutations are additive, we constructed targeted in-frame deletions of both genes in the S. aureus NCTC8325 background and measured TLR2/1 and TLR2/6 activity (Fig. 2). All deletion strains grew at rates statistically identical to the parent wild-type strain. Dilution series of heat-killed bacterial culture extracts were applied to TLR2/1-expressing reporter cells, and transcriptional activation of NF-κB was measured. Deletion of either or both genes in tandem decreased the signal equivalently, which could be restored to near-wild-type levels by plasmid back-complementation (Fig. 2A). With TLR2/6 assays, activity increased to the same extent in the single and double gene deletion constructs (Fig. 2B). The opposing TLR2 response indicates SAOUHSC_00822 and SAOUHSC_02761 are mutually required to swap TLR2 receptor ligand specificity. Since any combination of gene deletion alleles elicited equal changes in the TLR2 response, SAOUHSC_00822 and SAOUHSC_02761 are therefore codependent and not separate components of redundant Lpp *N*-acylation pathways.

### SAOUHSC_00822 and SAOUHSC_02761 alter the Lpp profile in *S. aureus*.

**FIG 3:**
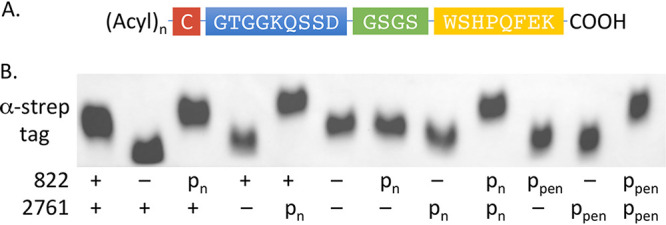
Immunoblot of SitC strep tag fragment expressed in S. aureus RN4220. (A) The SitC Lpp probe expressed by pLI50-sitC10AA is a 10-amino-acid SitC fragment with a C-terminal strep tag epitope and an *N-*acylated cysteine after being processed by Lgt and Lsp. Colors: red, N-terminal cysteine; blue, SitC fragment; green, linker; yellow, strep tag. (B) Total protein was extracted from lysostaphin treated cells and separated by SDS-PAGE before being transferred to a nitrocellulose membrane. The SitC probe was detected by immunoblotting with HRP α-strep tag conjugate. The SAOUHSC_00822 (822) and SAOUHSC_02761 genotypes (labeled 822 and 2761, respectively) are indicated, and results are representative of two separate experimental replicates. +, Present in chromosome; –, absent in chromosome; P_n_, plasmid with native chromosomal promoter from S. aureus; P_pen_, plasmid with constitutive promoter.

To correlate changes in TLR2 immunoassay data (Fig. 2) with Lpp structure, we introduced a plasmid encoding a fragment of the S. aureus SitC Lpp with a C-terminal strep tag probe under the control of the constitutive promoter *P*_tuf_ (Fig. 3A). The probe contained an N-terminal signal peptide for export, a lipobox for recognition by Lgt and Lsp for maturation, and the first 9 amino acids of SitC following the N-terminal cysteine linked to the strep tag epitope. The short probe length allowed separation of Lpp chemotypes with small mass differences by SDS-PAGE, including those due to total number of acyl chains. All S. aureus Lpp extracts produced a single homogenous band (Fig. 3B). In comparison to the wild type, the ΔSAOUHSC_00822 and ΔSAOUHSC_02761 constructs produced faster-migrating Lpp chemotypes that could be reverted back to wild type by plasmid back-complementation. Deletion of both genes (ΔSAOUHSC_00822 ΔSAOUHSC_02761) did not further change the Lpp profile, confirming a mutually nonredundant role for these genes in Lpp modification. To determine whether one candidate gene was needed for transcription of the other, we repeated the assay using a constitutive promoter (*P*_pen_). The extent of complementation was complete and identical to vectors with native promoters. The functional codependence of SAOUHSC_00822 and SAOUHSC_02761 is thus not based on transcriptional regulation in line with initial Northern blotting results (Fig. S1B).

### SAOUHSC_00822 and SAOUHSC_02761 constitute a novel lipoprotein *N*-acylation system (LnsAB) directing TA-Lpp synthesis in *S. aureus*.

**FIG 4:**
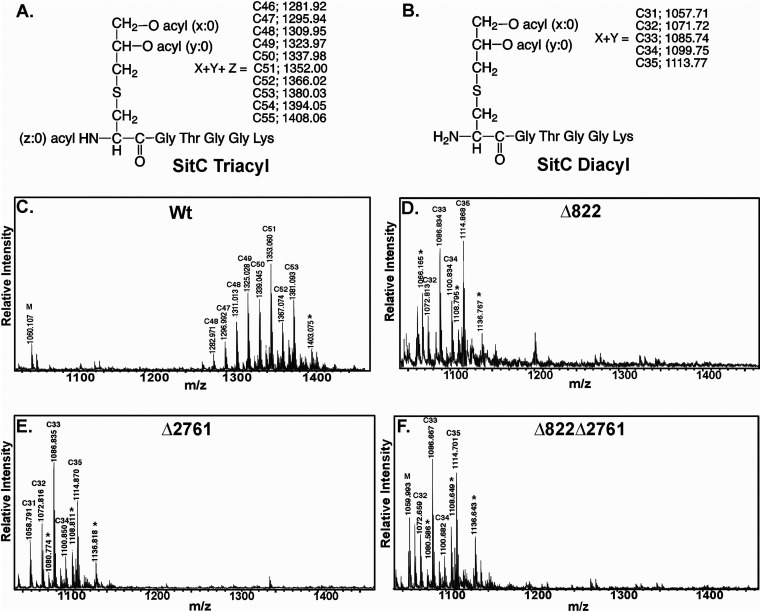
MALDI-TOF MS spectra of tryptic digest of S. aureus SitC Lpp. (A) The native structure of the N-terminal tryptic peptide of SitC Lpp in S. aureus is triacylated (TA-Lpp) and contains an amide-linked *N-*acyl chain. (B) Structure of the diacylated (DA-Lpp) N-terminal SitC tryptic lipopeptide. The calculated monoisotopic masses based on the sum total length of all acyl chains (C in carbon atoms) are indicated for TA-Lpp and DA-Lpp chemotypes. All acyl chains (ester-linked *sn*-1 *x*:0 and *sn-*2 *y*:0 plus amide-linked *z*:0) are assumed to be saturated. MALDI-TOF MS spectra of SitC N-terminal tryptic peptides in positive-ion mode were obtained from wild-type TM226 (Wt) (C), ΔSAOUHSC_00822 (D), ΔSAOUHSC_02761 (E), or the double-knockout ΔSAOUHSC_00822 ΔSAOUHSC_02761 strain (F). Sodiated adducts are represented by an asterisk (*). The α-CHCA matrix related peaks are labeled with an “M.” The MS/MS fragment ion spectra used to assign the acylation state of the N terminus (Fig. S2), and the MS spectra of the corresponding plasmid back-complemented strains (Fig. S3) are provided in the supplemental material.

To determine whether the mass shift observed by SDS-PAGE was indeed due to acylation state, native Lpps were extracted and analyzed by matrix-assisted laser desorption ionization–time of flight (MALDI-TOF) mass spectrometry (20, 43). The N-terminal lipopeptide spectrum from wild-type SitC yielded a characteristic series of ions differing by 14 U (-CH_2_-), consistent with a highly heterogeneous population of lipopeptides varying in total acyl chain (Fig. 4). The majority of the total signal could be assigned to the TA-Lpp chemotype, with the C51 chemotype being the dominant ion (M+H^+^ 1,353.06 U). Fragmentation of the C51 sodiated parent ion adduct (M+Na^+^ 1,375.06 U) produced a series of *N*-acyl dehydroalanyl signals separated by two methylene units (28 U), with C_18:0_ being the most abundant *N*-acyl fatty acid substitution (Fig. S2A). In contrast, deletion of SAOUHSC_00822 or SAOUHSC_02761 yielded MS spectra containing lower molecular mass lipopeptide signals between 1,057 and 1,137 U (Fig. 4D and E). The most abundant signals were C33 and C35 Lpp chemotypes (M+H^+^ 1086.7 U and 1114 u), nominally consistent with two acyl chains. Fragmentation of the C33 sodiated adduct (M+H^+^ 1,108.7 U) confirmed the canonical DA-Lpp acyl chain distribution with a diacyl glycerol moiety and a free α-amino terminal cysteine (Fig. S2B). Deletion of both genes resulted in an identical MS profile with similar acyl chain composition to single gene deletion mutants (Fig. 4F). TA-Lpp synthesis could be restored in all constructs by plasmid back-complementation (Fig. S3). Analysis of a set of extracts from a different Lpp (SAOUHSC_02699) yielded identical results as with SitC (Fig. S4). The MALDI-TOF MS and MS-MS results confirm that SAOUHSC_00822 and SAOUHSC_02761 have nonredundant roles in TA-Lpp formation. We have thus annotated this novel Lipoprotein *N*-acylation System as LnsA (SAOUHSC_00822) and LnsB (SAOUHSC_02761).

10.1128/mBio.01619-20.3 FIG S2 The MS-MS fragment ion spectra of the sodiated 1,375 U (A) and sodiated 1,108 U (B) tryptic peptide ions obtained from the wild-type S. aureus NCTC8325 TM226 strain and the ΔSAOUHSC_00822 ΔSAOUHSC_02761 deletion strain, respectively, that were used to assign N-terminal acylation states. The lipoprotein chemotype structures of tri-acylated (TA-Lpp, panel C) and diacylated (DA-Lpp, panel D) SitC are shown. All acyl chains are assumed to be saturated (z:0), and the mass heterogeneity due to differences in the length of the amide linked *N*-acyl chain are listed for TA-Lpp. Diagnostic fragment ions corresponding to the *y*-series amino acid sequence (pink) and the dehydroalanyl fragment (blue) are color coded. Primary MS spectra of samples are depicted in Fig. 4. Download FIG S2, TIF file, 1.0 MB. Copyright © 2020 Gardiner et al. 2020 Gardiner et al. This content is distributed under the terms of the Creative Commons Attribution 4.0 International license.

10.1128/mBio.01619-20.4 FIG S3 MALDI-TOF MS spectra of SitC N-terminal tryptic peptides isolated either from the plasmid back-complemented strains of ΔSAOUHSC_00822(p822) (A), ΔSAOUHSC_02761(p2761) (B), or the double knockout ΔSAOUHSC_00822 ΔSAOUHSC_02761(p822 p2761). Peaks are labeled by the sum number of total carbons in all fatty acid chains (C46 to C55) present in the SitC N-terminal lipopeptide (see Fig. 4 for chemotype structures). Addition of sodium is represented by an asterisk (*) and α-CHCA matrix associated peaks are labeled M. Primary MS spectra of isogenic parent strains are depicted in Fig. 4 of main text. Download FIG S3, TIF file, 2.7 MB. Copyright © 2020 Gardiner et al. 2020 Gardiner et al. This content is distributed under the terms of the Creative Commons Attribution 4.0 International license.

10.1128/mBio.01619-20.5 FIG S4 MALDI-TOF MS spectra of trypsinized SAOUHSC_02699 N-terminal lipopeptides isolated from either the S. aureus NCTC8325 strain TM226 wild-type (Wt) (A), the ΔSAOUHSC_00822 (B), the ΔSAOUHSC_02761 (C), and the double knockout ΔSAOUHSC_00822 ΔSAOUHSC_02761 (D) are shown. All gene deletion mutants reduce the wild-type mass peak family by masses consistent with the loss of a single acyl chain. Plasmid back-complementation restored the peak pattern to wild type in SAOUHSC_00822(p822) (E), in SAOUHSC_02761(p2761) (F), and in ΔSAOUHSC_00822 ΔSAOUHSC_02761(p822p2761) (G). The structures of triacyl (H) and diacyl (I) SAOUHSC_02699 N-terminal trytic lipopeptides along with the calculated monoisotopic masses are shown. The calculated masses are based on sum number of carbons in all fatty acid chains (ester-linked *sn*-1 *x*:0 and *sn-*2 *y*:0 plus amide-linked *z*:0), which are assumed to be saturated. Sodium adducts are represented by an asterisk (*), potassium adducts by “k,” and α-CHCA matrix-related peaks by “M.” Download FIG S4, TIF file, 2.8 MB. Copyright © 2020 Gardiner et al. 2020 Gardiner et al. This content is distributed under the terms of the Creative Commons Attribution 4.0 International license.

### N-terminal Lpp modification attenuates detection by TLR2/1/6.

**FIG 5:**
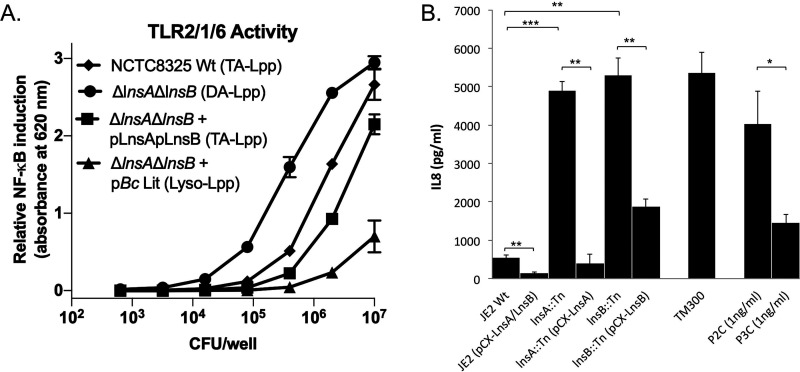
TLR2/1/6 activity and IL-8 induction in response to S. aureus Lpp chemotypes. (A) Relative NF-κB induction in HEK-Blue reporter cells (hTLR2/1/6) expressing both TLR2/1 and TLR2/6 receptor complexes were stimulated with either wild-type S. aureus NCTC8325, Δ*lnsA*Δ*lnsB*, Δ*lnsA*Δ*lnsB* + pLnsA pLnsB, or Δ*lnsA*Δ*lnsB* + p*Bc*Lit. The Lpp chemotype produced by each strain is indicated parenthetically. The data are from experiments conducted in triplicate and the error bars show standard deviation values. TA-Lpp, triacylated; DA-Lpp, diacylated; Lyso-Lpp, lyso-form. (B) IL-8 production (pg/ml) was measured after an 18-h stimulation of HEK-TLR2 cells with S. aureus USA300 wild-type JE2, JE2(pCX-LnsA/LnsB), Tn::*lnsA* (NE536), Tn::*lnsA*(pCX-LnsA), Tn::*lnsB* (NE407), Tn::*lnsB*(pCX-LnsB), and *S. carnosus* TM300. The MOI was 2. Tripalmitoylated (P3C) and dipalmitoylated (P2C) CSKKKK synthetic lipopeptides were used as controls for TLR2/1 and TLR2/6 activity, respectively. The experiments were conducted in triplicate and performed more than three times. The error bars indicate standard deviation values. Statistical significances were calculated by using Student *t* tests (*, *P* < 0.05; **, *P* < 0.01; ***, *P* < 0.001).

With the discovery of LnsAB, we could now directly compare the TLR2-stimulating potential of DA-Lpp, TA-Lpp, and lyso-Lpp chemotypes in the same S. aureus genetic background. Reporter cells expressing TLR2/1/6 (capable of binding both DA-Lpp and TA-Lpp ligand) were challenged with heat-killed bacterial cells, and the NF-κB transcriptional activation was measured (Fig. 5A). A clear hierarchy was observed in the bacterial cell count needed to reach half-maximal activation (EC_50_). The EC_50_ of S. aureus expressing DA-Lpp was >100-fold lower in comparison to an isogenic lyso-Lpp forming strain carrying *lit* from B. cereus. The EC_50_ for wild-type S. aureus synthesizing TA-Lpp was intermediate, increasing the EC_50_ from DA-Lpp by 10-fold. Lpp chemotype potency for TLR2 activation is hence ordered (DA-Lpp > TA-Lpp > lyso-Lpp), demonstrating how *Firmicutes* Lpp N-terminal modification systems can alter TLR2 detection over more than 2 orders of magnitude.

In order to translate changes in the NF-κB transcriptional response into proinflammatory cytokine secretion, we repeated the assay using *lnsAB* Tn insertion mutants in the contemporary clinical isolate S. aureus USA300 [NE536(Tn*0780*)-LnsA and NE407(Tn*2405*)-LnsB initially identified in Fig. 2A]. If both S. aureus genes direct TA-Lpp formation, the immune-stimulating activity in *lnsAB* deletion mutants making only DA-Lpp should be significantly increased as well (Fig. 4). Immune stimulation was monitored by the production of IL-8. The wild-type JE2 parent produced only about 500 pg/ml, whereas in the two Tn mutants NE536(*lnsA*::Tn) and NE407(*lnsB*::Tn) the IL-8 production was ∼10 times higher (Fig. 5B). When back-complemented in either NE536(pCX-LnsA) and NE407(pCX-LnsB), the IL-8 production was decreased. Conversely, when both genes were coexpressed in JE2 (pCX-LnsA/LnsB), IL-8 production was even further decreased, indicating high-level *N*-acyl transferase activity further shifted the Lpp chemotype population in favor of the weaker TA-Lpp TLR2 agonist. The cytokine response is in complete agreement with the NF-κB transcriptional activation data. It has previously been shown that *N*-acetyl Lpp induces higher immune stimulation than TA-Lpp in *S. carnosus* (23), and direct comparison of *S. carnosus* to DA-Lpp-producing S. aureus *lnsA* or *lnsB* mutants demonstrated nearly equivalent activity. Thus, staphylococci forming *N*-acetyl or DA-Lpp are higher TLR2 activating agonists than their TA-LPP counterparts.

## DISCUSSION

There is much naturally occurring structural diversity among Lpp chemotypes in *Firmicutes* (19). Unlike in Gram-negative bacteria, deletion of the core Lpp biosynthetic genes (Lgt and Lsp) in monoderm Gram-positive bacteria such as S. aureus induces a subtle phenotype in rich media (44), and a robust phenotype specifically attributable to the Lpp N-terminal acylation state has yet to be reported. We therefore utilized loss of TLR2/1 activation to identify bacterial mutants with changes in the Lpp N-terminal acylation state. We screened two separate S. aureus Tn insertion libraries and, surprisingly, identified two previously unknown genes necessary for TA-Lpp production that we have now named LnsA (SAOUHSC_00822) and LnsB (SAOUHSC_02761). Both *lnsAB* genes are absolutely required for TA-Lpp formation in S. aureus as determined by the Lpp SDS-PAGE profiles (Fig. 3) and MALDI-TOF mass spectrometry (Fig. 4; see also Fig. S4). LnsAB share no similarity with the two other known Lpp *N*-acylating enzymes in either primary amino acid sequence or mechanism. The apolipoprotein *N*-acyl transferase (Lnt) in Gram-negative bacteria utilizes the *sn-*1 acyl chain of phosphatidylethanolamine as an *N*-acyl chain donor (45), while Lit intramolecularly transfers the *sn-*2 acyl group of DA-Lpp to the α-amino terminus to form lyso-Lpp (46). The acyl chain source for LnsAB is currently unknown. While other genes may be needed to make the acyl donor, these genetic determinants are not unique to S. aureus since TLR2/1 agonist activity was conferred to L. monocytogenes by integrating just *lnsAB* into the genome (Fig. 1C).

**FIG 6:**
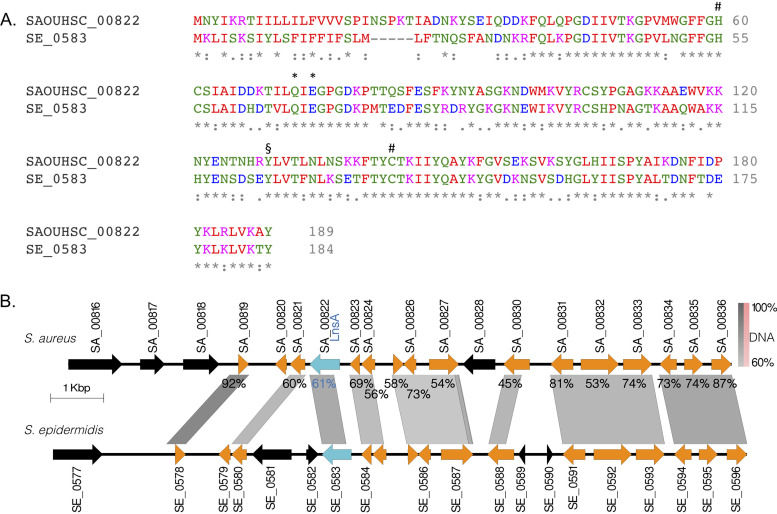
Bioinformatic analysis of LnsA orthologs in staphylococci. (A) An alignment of the characterized LnsA (SAOUHSC_00822) protein from *S. aureus* with the SE_0583 ortholog from S. epidermidis ATCC 12228 was created using Clustal Ω (97). The S. aureus and S. epidermidis proteins share 61% amino acid identity. The genome of *S. carnosus* TM300 has no open reading frames with significant similarity. The four important catalytic residues in PPNEs equivalent to those in papain are indicated: invariant histidine/cysteine dyad residues (#), the NlpC/P60 protein superfamily tyrosine signature residue (§), and two candidates for the third active site polar residue (*) (47). (B) The genetic loci for each staphylococcus strain were aligned by DNA sequence homology centered around the respective LnsA open reading frame (LnsA from S. aureus and SE_0583 in S. epidermidis ATCC 12228) and are indicated in blue. Regions with DNA homology (% identical base pairs indicated by bar scale) are indicated in gray. Genes common to both species are indicated in orange, and genes without orthologs in the genomic region shown are black. The percent amino acid sequence identity is indicated for each similar locus tag gene pair. Plot was constructed using EasyFig (98).

TA-Lpp formation in S. aureus requires both LnsA and LnsB, but at present their respective roles in *N*-acylation are unknown. The top five proteins with homology to LnsA (YiiX [2IF6], 18% identity; BCE_A0238 [3KW0] [47], 14% identity; HRASLS-2/PLA_1/2_-2 [4DPZ] [48], 13% identity; HRASLS-4/TIG3 [2LKT] [49], 14% identity; and HRASLS-3/H-REV107 [50], 15% identity) as predicted by HHPred (51) are all enzymes in the papain-like NlpC/P60 superfamily (41). The NlpC/P60 superfamily consists of four main families (P60-like, AcmB/LytN-like, YaeF/YiiX-like, and LRAT-like) that can be further grouped into the canonical CPNE (P60-like and AcmB/LytN-like) and permuted PPNE (YaeF/YiiX-like and LRAT-like) subsets (47). Both CPNE and PPNE families have similar tertiary structures, but in PPNEs, the residue order of the active site triad (His/Cys/polar) is swapped (41, 47). The cysteine/histidine dyad needed to form the covalent acyl-thioster substrate-enzyme intermediate in the NlpC/P60 superfamily active site is invariant (52, 53). CPNEs are common in prokaryotes, acting as extracellular hydrolases, peptidases, and amidases that remodel peptidoglycan (54, 55). The functions of the bacterial PPNEs (YaeF/YiiX-like) are poorly understood (47). Although the overall sequence similarity with LnsA is modest, all five HHPred homology hits are PPNEs, and the corresponding permuted active site residues in LnsA can be inferred (Fig. 6A). Interestingly, YiiX endogenously cocrystallized with a fatty acid in a hydrophobic S1 binding pocket that appears to be conserved in PPNEs (47). Unlike the prokaryotic YaeF/YiiX-like PPNE subfamily, the LRAT (lecithin:retinol acyl transferase-like enzymes) are a diverse group of enzymes in vertebrates with well-established functions in glycerophospholipid remodeling metabolism (48). Three of the top five proteins with similarity to LnsA are H-RAS-like suppressor (HRASLS) family members, a group of enzymes with shared phospholipase A_1/2_ (PLA_1/2_) hydrolase activity, as well as *O*- and *N*-acyltransferase activity (56, 57). HRASLS-2, HRASLS-3, and HRASLS-4 all have PLA_1/2_ hydrolase activity *in vitro* and, in the case of HRASLS-2/HRASLS-3, also have *N*-acyltransferase activity that utilizes a phosphatidylcholine acyl chain donor to convert phosphatidylethanolamine into *N*-acyl phosphatidylethanolamine (57–59). The latter activity, in particular, has strong parallels with the biochemistry required for Lpp *N*-acylation and makes LnsA the more obvious catalytic candidate for acyl transferase activity. Among the three *Staphylococcus* species with experimentally characterized Lpp structures (S. aureus TA-Lpp, S. epidermidis TA-Lpp, and *S. carnosus N-*acetyl-Lpp), LnsA orthologs are only present in the genomes of S. aureus and S. epidermidis, with a common genome synteny and sequence conservation at both the DNA and the protein level (61% identity, Fig. 6B).

Aside from being absolutely required for TA-Lpp formation (Fig. 3 and 4) and TLR2/1 specific detection in S. aureus (Fig. 1B, 2, and 5) or L. monocytogenes when heterogeneously expressed (Fig. 1C), the function of LnsB in Lpp *N*-acylation is much more speculative than LnsA. LnsB does have very weak similarity to the CAAX prenyl protease from the archaeal methanogen Methanococcus maripaludis (Rce1 [4CAD_C] [60], 14% identity) and the APH-1A subunit of human γ-secretase (APH-1A [5A63_C] [61], 7% identity). Both of these integral membrane proteins are part of the CAAX protease and bacteriocin-processing enzyme (CPBP) family (62), a large class of enzymes encompassing metalloproteases and other integral membrane proteins with poorly defined cellular function. Many bacteria encode multiple CPBPs, with S. aureus NCTC8325 containing at least six other CPBP enzymes in addition to LnsB (62). Of these, MroQ is a suspected protease that processes auto-inducing peptide (63, 64). There is phenotypic evidence for roles of four other staphylococcal CPBPs in maintaining cell envelope integrity (65) and in the expression of cell wall-attached surface proteins with YSIRK peptide signals (66). It is apparent from these studies, however, that the cellular functions are not entirely overlapping, and at least one (SAOUHSC_02611/LyrA/SpdC) is almost certainly not a protease since key catalytic residues are absent (67). A similar analysis of catalytic motifs in LnsB shows considerable divergence from all CPBP-subfamily signature motifs as well, particularly in motif 4 (Fig. S5A). The similarity of LnsB to CAAX proteases, albeit without conservation of catalytic residues, suggests the CPBP fold could have been coopted for a noncatalytic chaperone role analogous to that suggested for the APH-1 subunit of γ-secretase (68). APH1 has low sequence conservation of CPBP catalytic residues like LnsB (Fig. 6A) and no standalone proteolytic activity. Instead, APH1 is proposed to present protein substrate to the presenilin protease subunit core for hydrolysis within the γ-secretase complex (69). Whether LnsB physically associates with LnsA in a complex, contributes any catalytic residues to the active site, or interacts with Lpp substrates remains to be determined. Alternative models where LnsB makes a novel acyl donor that is used by LnsA or where LnsB is indirectly required to process or stabilize LnsA cannot be ruled out.

10.1128/mBio.01619-20.6 FIG S5 Bioinformatic analysis of LnsB and CPBP family proteins from staphylococci. (A) Three of the main amino acid sequence motifs identified in CPBP family proteins are shown for LnsB from S. aureus NCTC8325 (Sa), along with the closest orthologs in S. epidermidis ATCC 12228 (Se2027) and *S. carnosus* TM300 (SCA_1941). Rce1 is a CAAX prenyl protease from Methanococcus maripaludis identified as having similarity with LnsB using HHPred. Amino acid numbering is shown for each protein. Motif signatures thought to be important for catalysis from each of the CPBP subfamilies are indicated for comparison (62). x, any amino acid, usually hydrophobic; s, small amino acid; B, asparagine or aspartate. Superscript letters: a, motif signature numbering; b, CPBP subfamilies; c, archeal strains classified within DUF2324 (all according to reference 3). (B) The genetic loci for each staphylococcus strain were aligned by DNA sequence homology centered on the respective CPBP open reading frame (LnsB from S. aureus, SE_2027 in S. epidermidis ATCC 12228, and SCA_1941 in *S. carnosus*) and are indicated in blue. Regions with DNA homology (% identical base pairs indicated by bar scale) are indicated in gray, while inverted DNA regions are indicated in pink. Genes common to all species are indicated in purple, those common to all but inverted are indicated in yellow, those only between S. aureus and S. epidermidis are indicated in orange, and those only between S. epidermidis and *S. carnosus* in are indicated green. Genes without orthologs in the genomic region shown are indicated in black. The percent amino acid sequence identity is indicated for each similar locus tag gene pair. Plot was constructed using EasyFig (98). Download FIG S5, TIF file, 1.8 MB. Copyright © 2020 Gardiner et al. 2020 Gardiner et al. This content is distributed under the terms of the Creative Commons Attribution 4.0 International license.

There are also substantial differences in gene content and arrangement between the S. aureus LnsB genomic locus and the corresponding positions in both S. epidermidis and *S. carnosus* genomes. Genomic synteny in the LnsB loci between the staphylococci strains is highly mosaic, suggesting possible species-specific recombination or even horizontal acquisition events (Fig. S5B). While S. aureus (TA-Lpp) has common flanking genes with S. epidermidis (TA-Lpp) and *S. carnosus* (*N-*acetyl Lpp) on only one side, they are inverted with respect to LnsB (Fig. S5B). Gene architecture in S. epidermidis is intermediate and shares certain features with both genomes. Curiously, an LnsB-like CPBP open reading frame in the same position is present in all three genomes, including in the *N*-acetyl Lpp forming *S. carnosus* genome (SCA_1941). The overall sequence similarity between all three CPBP proteins is much lower (25 to 28% identical) though, in comparison to neighboring genome segments, which is inconsistent with expectations for simple species-driven genetic drift. Once more, no homology can be detected between the CPBP open reading frames at the DNA level, suggesting functional divergence as well as a possibly independent origin for all three genes. As with LnsB from S. aureus, the S. epidermidis ortholog (SE_2027) does not have many of the essential CPBP catalytic motifs and thus likely functions in Lpp *N*-acylation as well (Fig. S5A). The *S. carnosus* CPBP gene (SCA_1941) in comparison has all the CBPB canonical signature residues, including a completely intact motif 4. The sequence divergence may reflect the difference in catalytic activity (Lpp *N*-acetylation versus *N*-acylation) or, more likely, that SCA_1941 is not a functional LnsB ortholog and that the seemingly conserved synteny is due to genome rearrangement events.

The identification of LnsAB expands the catalog of known TLR2 recognition factors for staphylococci (Fig. 5). The TLR2-stimulating potential between different *Firmicutes*, and even within the same species, can vary significantly (11). In S. aureus, differences in total Lpp gene content, capsular polysaccharide thickness, and autolysis rates can all attenuate ligand release and accessibility (70). Phenol soluble modulins produced by S. aureus are surfactant-like small peptides that enhance release of Lpp-loaded extracellular vesicle release and in turn alter TLR2 responses (71). Disparity also stems from TLR2 antagonizing factors. The lipoylated E2 subunit of the pyruvate dehydrogenase complex suppresses TLR2/1 activity (72), while levels of secreted lipases that degrade shed Lpp is subject to lysogenic bacteriophages (73). An additional factor is the Lpp chemotype itself, which can indirectly attenuate the TLR2 response by acting through any of the above mechanisms, or more simply by altering ligand specificity and/or affinity at the respective TLR2 receptor complexes as demonstrated here. It should be noted that in staphylococci there are no other compounds described that activate TLR2 and Lpp is the dominant immunobiologically active ligand (23, 74).

Although TLR2 attenuation is clearly an outcome of chemotype conversion from DA-Lpp to TA-Lpp, there is likely an overarching selective pressure for Lpp *N*-modification that supersedes immune evasion. Noncommensal strains such as *S. carnosus* and environmental strains such as B. subtilis have Lpps modified with *N*-acetyl groups. Analogous *N*-acetyl amino terminal tailoring has even been reported in archaea (75). Some archaea express Lpp-like, membrane-associated proteins with a characteristic lipobox preceding an invariant cysteine residue as in bacteria, except that they are thought to be modified with diphytanoyl glycerol diether lipid (76). TLR2 immunomodulation is an unlikely motivation for N-terminal tailoring in any of these cases. One possible clue regarding a broader, universal selective pressure operating outside the host TLR2-bacterial niche is offered by the recent discovery of an Lit2 paralog in L. monocytogenes (21). The *lit2* gene is embedded within a copper resistance operon on either a transposon or transmissible plasmid in select environmental isolates. Like chromosomally encoded Lit, Lit2 converts DA-Lpp to lyso-Lpp but is specifically induced by copper ions. It was suggested that Lpp *N*-acylation may help prevent copper coordination at the membrane surface, limiting its uptake or oxidative damage from copper-mediated redox cycling (21). In E. coli, copper exposure induces DA-Lpp accumulation and Lpp outer membrane trafficking defects (77), while intracellular copper accumulates in Lnt depletion strains (78). Copper as a selective pressure would help explain the genesis of novel Lpp acylation systems such as LnsAB and more broadly the Lpp chemotype heterogeneity observed in *Firmicutes*. Copper selective pressure has grown in step with environmental oxygenation levels that increase copper bioavailability (79). Contemporary selection for copper resistance determinants has also arisen from copper’s widespread current use as an antimicrobial agent (80). Assuming that the core Lgt-Lsp pathway was initially established in prokaryotes and that selective pressure for Lpp *N*-acylation arose after establishment of the various lineages, different bacterial species would have subsequently acquired *N*-acylation systems independently from each other. A particularly intriguing theory proposes modern *Firmicute* monoderm lineages independently arose at multiple times from a common diderm ancestor through loss of genes directing the biogenesis of the second outer membrane (81, 82). If a common ancestral Lpp *N*-acylation system was lost in tandem, multiple independent Lpp *N*-acylating gene reacquisition events would have followed. In either case, the Lpp *N*-acylation system diversity exemplified by LnsAB has provided a ready-made genetic reservoir to modulate TLR2-mediated immunodetection among *Firmicutes*.

## MATERIALS AND METHODS

### Bacterial strains and growth conditions.

All E. coli strains were grown in lysogeny broth-Miller medium (LB), while S. aureus and L. monocytogenes strains were grown in tryptic soy broth (TSB) at 37°C in baffled flasks (3-to-1 flask to culture ratio) with continuous aeration at 250 rpm unless indicated otherwise. For cytokine production assays, S. aureus strains were cultivated in basic medium (BM; 1% soy peptone, 0.5% yeast extract, 0.5% NaCl, 0.1% glucose, and 0.1% K_2_HPO_4_ [pH 7.2]) at 37°C under continuous shaking at 150 rpm. Antibiotic resistance markers were selected with carbenicillin (100 μg/ml), kanamycin (30 μg/ml), spectinomycin (50 μg/ml), chloramphenicol (20 μg/ml in E. coli, 10 μg/ml for plasmid or 5 μg/ml for integrated marker in S. aureus, and 3 μg/ml in L. monocytogenes), and erythromycin (5 μg/ml) where appropriate. The S. aureus NCTC8325 HG003 transposon library, built in the derivative strain TM226 (30), and the Nebraska Transposon Mutant Library (NTML), built in S. aureus strain USA300 (83), were cultured in 96-well microplates with buffered TSB (50 mM HEPES [pH 7.4]) without shaking in a humidified environment at 37°C. For pET22-based vectors, gene expression was induced using 1 mM IPTG (isopropyl-β-d-thiogalactopyranoside). For expression of xylose-inducible genes encoded in plasmid pCX30, BM medium was supplemented with 10 μg/ml chloramphenicol, and glucose was substituted by 0.5% (wt/vol) xylose. Generation times were measured in 96-well microplates in TSB media incubated at 37°C. All strains and plasmids are listed in Table 1.

**TABLE 1:** Bacterial strains and plasmids used in this study

Strain or plasmid	Relevant genotype and/or phenotype^*a*^	Source or reference
Strains		
*E. coli*		
BW25113	*E. coli* K-12 wild type; Δ(*araD araB*)*567* Δ*lacZ4787*(::*rrnB-3*) *λ^−^ rph-1* Δ(*rhaD-rhaB*)*568 hsdR514*	CGSC7636^*b*^
KA327	BW25113 Δ*lpp*	20
KA548	*lpp*::Cm^r^ + pCL25 ori-pKD4 kanR-lppK58A-strep; synthesizes triacylated Lpp that cannot be covalently cross-linked to peptidoglycan	20
KA775	*lpp*::Cm^r^ *lnt*::Spec^r^ + pKA522-PA3286 (pCL25 ori-kanR-lppK58A strep-PA3286) (spontaneous suppressor mutant); synthesizes diacylated Lpp that cannot be covalently cross-linked to peptidoglycan	46
*S. aureus*		
TM226	*S. aureus* HG003 NCTC8325 ϕ11::FRT	30
Tn 16C2	TM226 Tn insertion 18-bp upstream of SAOUHSC_02761 start codon, Erm^r^	This study
Tn 32F1	TM226 Tn insertion in amino acid 114 of SAOUHSC_02761, Erm^r^	This study
JG1299	TM226 *lsp*::Tn, Erm^r^	This study
JG1300	TM226 *lgt*::Tn, Erm^r^	This study
JG1497	TM226 ΔSAOUHSC_02761	This study
JG1498	TM226 ΔSAOUHSC_00822	This study
JG1499	TM226 ΔSAOUHSC_00822 ΔSAOUHSC_02761	This study
TXM1515	JG1497 + pCN59 *P*_nat_ SAOUHSC_02761, Erm^r^	This study
TXM1516	JG1498 + pCN59 *P*_nat_ SAOUHSC_00822, Erm^r^	This study
TXM1523	JG1499 + p*Bc* Lit, Cm^r^; synthesizes lyso-form Lpps, Cm^r^	This study
TXM1529	JG1499 + pCN59 *P*_nat_ SAOUHSC_02761 SAOUHSC_00822, Erm^r^	This study
RN4220	*S. aureus* NCTC8325 restriction negative, prophage cured	94
TXM1485	RN4220 ΔSAOUHSC_02761	This study
TXM1486	RN4220 ΔSAOUHSC_00822	This study
TXM1500	RN4220 ΔSAOUHSC_02761 ΔSAOUHSC_00822	This study
TXM1510	TXM1486 + pCN59 *P*_nat_ SAOUHSC_00822, Erm^r^	This study
TXM1511	TXM1485 + pCN59 *P*_nat_ SAOUHSC_02761, Erm^r^	This study
TXM1528	TXM1500 + pCN59 *P*_nat_ SAOUHSC_02761 SAOUHSC_00822, Erm^r^	This study
TXM1577	TXM1500 + pLI50-sitC10AA, Cm^r^	This study
TXM1578	TXM1486 + pLI50-sitC10AA, Cm^r^	This study
TXM1579	RN4220 + pLI50-sitC10AA, Cm^r^	This study
TXM1580	TXM1577 + pCN59 *P*_nat_ SAOUHSC_02761 SAOUHSC_00822, Cm^r^ Erm^r^	This study
TXM1581	TXM1578 + pCN59 *P*_nat_ SAOUHSC_00822, Cm^r^ Erm^r^	This study
TXM1582	TXM1583 + pCN59 *P*_nat_ SAOUHSC_02761, Cm^r^ Erm^r^	This study
TXM1583	TXM1485 + pLI50-sitC10AA, Cm^r^	This study
TXM1584	TXM1577 + pCN59 *P*_nat_ SAOUHSC_00822, Cm^r^ Erm^r^	This study
TXM1585	TXM1577 + pCN59 *P*_nat_ SAOUHSC_02761, Cm^r^ Erm^r^	This study
TXM1586	TXM1577 + pCN59 *P*_pen_ SAOUHSC_00822, Cm^r^ Erm^r^	This study
TXM1587	TXM1577 + pCN59 *P*_pen_ SAOUHSC_02761, Cm^r^ Erm^r^	This study
TXM1588	TXM1577 + pCN59 *P*_pen_ SAOUHSC_02761 SAOUHSC_00822, Cm^r^ Erm^r^	This study
JE2	*S. aureus* USA300 wild-type cure of endogenous plasmids	BEI
NE536	SAUSA300_0780::Tn, Erm^r^	BEI
NE407	SAUSA300_2405::Tn, Erm^r^	BEI
*L. monocytogenes*		
Wild type	*L. monocytogenes* Li2 ATCC 19115	ATCC^*c*^
TXM1530	*att*::pPL2-P_pen_ SAOUHSC_00822	This study
TXM1531	*att*::pPL2-P_pen_ SAOUHSC_02761	This study
TXM1532	*att*::pPL2-P_pen_ SAOUHSC_02761 and SAOUHSC_00822	This study
*S. carnosus*		
Wild type	*S. carnosus* strain TM300	95
		
Plasmids^*d*^		
pLI50	*E. coli*-*S. aureus* shuttle vector, Carb^r^ Cm^r^	86
pLI50-PpenGfpmut2	pLI50 with *P*_pen_ insert to control expression; Carb^r^ Cm^r^	96
pLI50-sitC10AA	pLI50-*P*_tuf_ SitC N terminus 10-amino-acid fragment-strep tag TT	This study
pCN59	*E. coli*-*S. aureus* shuttle vector, Carb^r^ Erm^r^	87
pGKM1456	pCN59 *P*_nat_ SAOUHSC_00822, Carb^r^ Erm^r^	This study
pTXM1505	pCN59 *P*_nat_ SAOUHSC_02761, Carb^r^ Erm^r^	This study
pTXM1508	pCN59 *P*_pen_ SAOUHSC_00822, Carb^r^ Erm^r^	This study
pTXM1509	pCN59 *P*_pen_ SAOUHSC_02761, Carb^r^ Erm^r^	This study
pTXM1512	pCN59 *P*_pen_ SAOUHSC_02761 SAOUHSC_00822, Carb^r^ Erm^r^	This study
p*Bc*Lit	pLI50 *P*_pen_ *Bacillus cereus lit* gene, Carb^r^ Cm^r^	This study
pTXM1524	pCN59 *P*_nat_ SAOUHSC_02761 SAOUHSC_00822, Carb^r^ Erm^r^	This study
pPL2	*E. coli* shuttl*e-L. monocytogenes* integration vector, Cm^r^	88
pTXM1525	pPL2*-P*_pen_ SAOUHSC_00822, Cm^r^	This study
pTXM1526	pPL2*-P*_pen_ SAOUHSC_02761, Cm^r^	This study
pTXM1527	pPL2*-P*_pen_ SAOUHSC_02761 SAOUHSC_00822, Cm^r^	This study
pTXM908	pBBR1 ori *P*_Kan_-*lolCDE*	46
pCX30	pC194 ori, *P*_xyl_ to control expression, Cm^r^	89
pCX-LnsA/LnsB	pCX30 *P*_xyl_ LnsA (SAUSA300_0780) and LnsB (SAUSA300_2405), Cm^r^	This study
pCX-LnsA	pCX30 *P*_xyl_ LnsA (SAUSA300_0780), Cm^r^	This study
pCX-LnsB	pCX30 *P*_xyl_ LnsB (SAUSA300_2405), Cm^r^	This study

a Resistance phenotypes: Carb^r^, carbenicillin; Cm^r^, chloramphenicol; Kan^r^, kanamycin; Erm^r^, erythromycin resistance; Spec^r^, spectinomycin.

b Strain CGSC7636 at the Coli Genetic Stock Center (CGSC).

c American Type Culture Collection.

d TT, transcriptional terminator.

### Construction of bacterial deletion strains and plasmids.

Gene deletions were constructed using the temperature-sensitive shuttle vector pKFC in S. aureus, as previously described (84, 85). Plasmids were assembled from two separate 1-kb DNA fragments flanking the gene targeted for deletion and obtained by PCR. About 10 coding triplets from both ends of the targeted gene were retained to create nonpolar, in-frame gene deletions. Fragments were assembled using the In-Fusion HD cloning kit (TaKaRa Bio) and transformed into restriction negative S. aureus RN4220 by electroporation. Plasmids were then isolated and also transformed into strains TM226 and JE2 for integration and outcross in these genetic backgrounds. Deletion alleles were confirmed by PCR using primers annealing outside the targeted locus (Fig. S6). Complementation plasmids expressing SAOUHSC_00822, SAOUHSC_02761, or both genes in tandem were built from fragments amplified from S. aureus NCTC8325 genomic DNA and cloned into pLI50 (86), pCN59 (87), or pET22 (Novagen) using the same method and verified by sequencing. Expression of S. aureus genes in L. monocytogenes was achieved by integration into the chromosome using the pPL2 integration vector (88). The xylose-inducible pCX30 based complementation vectors (89) were constructed by PCR amplifying the two genes (SAUSA300_0780 and SAUSA300_2405) from S. aureus USA300 genomic DNA. The PCR inserts were cloned into pCX30 linearized with BamHI and SmaI using Hi-Fi DNA Assembly Master Mix (New England Biolabs). The resulting plasmid was transformed into *S. carnosus* TM300 by electroporation. Plasmid-harboring colonies were picked and verified by DNA sequencing. The correct plasmid was then transformed into wild type [JE2 (pCX-LnsA/LnsB)]. The Tn mutant strains from the NTML library [NE536(pCX-LnsA) and NE407(pCX-LnsB)] were complemented using single-gene-expressing vectors constructed in the same way. All primers are listed in Table S1 in the supplemental material.

10.1128/mBio.01619-20.1 TABLE S1 Primers used in this study. Download Table S1, DOCX file, 0.03 MB. Copyright © 2020 Gardiner et al. 2020 Gardiner et al. This content is distributed under the terms of the Creative Commons Attribution 4.0 International license.

10.1128/mBio.01619-20.7 FIG S6 PCR amplification of all relevant lipoprotein related loci in the S. aureus RN4220 (A) and in S. aureus NCTC8325 strain TM226 (B) using primers (∼1 kb up- and downstream of the coding region) flanking either SAOUHSC_02761 (*lnsB*) or SAOUHSC_00822 (*lnsA*) confirmed the anticipated chromosomal genotypes. The presence of complementing plasmid was likewise detected by PCR using plasmid-specific primer pairs. Plasmid, pCN59 vector check primers; plasmid*, pLI50 vector check primers. Download FIG S6, TIF file, 0.5 MB. Copyright © 2020 Gardiner et al. 2020 Gardiner et al. This content is distributed under the terms of the Creative Commons Attribution 4.0 International license.

### Transposon library screening for TLR2 activity.

Screens for Tn mutants modulating TLR2 signaling were conducted with two libraries: (i) our in-house Tn library constructed and described by Santiago et al. using S. aureus NCTC8325 strain TM226 (30) and (ii) the NTML built in S. aureus strain USA300 JE2 (83). For the TM226 transposon library, the glycerol stock Tn pool was diluted and streaked to single colonies on tryptic soy agar (TSA; 5 μg/ml erythromycin) and incubated overnight at 37°C. The next morning, individual colonies were inoculated into buffered TSB (200 μl/well with 5 μg/ml erythromycin) distributed in 96-well plates. Controls were included on each microplate (S. aureus RN4220 wild type and medium only). Microplates were incubated for 18 h at 37°C without agitation before bacterial cultures were resuspended by pipetting up and down three times. Aliquots (20 to 50 μl) were transferred to a 96-well PCR plate, and bacteria were heat killed by incubation at 58°C for 1 h. The NTML screen was conducted in an identical manner, except that the growth microplates were inoculated with 5 μl of thawed glycerol stocks from the prearrayed library stock plate. Heat-killed bacterial extracts were stored at 4°C until use.

Human embryonic kidney 293 cells (HEK-Blue hTLR2-TLR1; Invivogen) with endogenous TLR1 and TLR6 deleted and stably transfected with TLR2, TLR1, and an NF-κB responsive secreted alkaline phosphatase reporter gene were cultured as recommended by the manufacturer and recently described (90). On the day of the assay, ∼70% confluent HEK-Blue hTLR2-TLR1 cells were washed with 1× phosphate-buffered saline (PBS), harvested by centrifugation, counted, and diluted to the recommended final concentration of ∼280,000 cells/ml in Dulbecco modified Eagle medium (DMEM) without selective antibiotics. To each well containing 190 μl of cell culture medium, 10 μl of the heat-killed bacterial extract was added. Microplates were then incubated at 37°C in a 5% CO_2_ atmosphere for 44 h (for TLR2/1 assays), which pilot studies determined to be optimal for the largest dynamic assay range. Secreted alkaline phosphate was assayed as described previously (90), with minor modifications. Aliquots (20 μl) of supernatant were removed and added to 180 μl of QuantiBlue detection reagent (Invivogen), followed by incubation for 4 h before the absorbance was measured at 620 nm. Defined TLR2 Lpp ligands prepared from E. coli cells expressing either TA-Lpp (KA548) or DA-Lpp (KA775) were used as stimulation controls.

Primary Tn mutant hits were struck to single colonies and the decrease in TLR2/1 activity assay results confirmed. These samples were then tested for retention of TLR2/6 specific activity using HEK-Blue hTLR2-TLR6 cells (Invivogen) as described above except cells were stimulated for 20 h. All genotypes were checked and confirmed by PCR using primers targeting the *lgt*, *lsp*, and candidate *N*-acylation genes in the prearrayed NTML (Fig. S7). For the TM226 library (Fig. S1E), Tn insertion sites were mapped by inverse PCR of circularized gDNA fragments, as described elsewhere, except that *Taq*αI (New England Biolabs) was used for DNA restriction (91).

10.1128/mBio.01619-20.8 FIG S7 PCR amplification of all relevant lipoprotein related loci in the S. aureus USA300 JE2 NTML library using primers (∼1 kb up- and downstream of the coding region) flanking either *lgt*, *lsp*, SAUSA300_2405 (*lnsB*), or SAUSA300_0780 (*lnsA*) confirmed the anticipated genotypes. Download FIG S7, TIF file, 0.3 MB. Copyright © 2020 Gardiner et al. 2020 Gardiner et al. This content is distributed under the terms of the Creative Commons Attribution 4.0 International license.

### TLR2 dose-response HEK-Blue reporter assays.

The TLR2 stimulating activity of Lpp *N*-acylation mutants was assayed using HEK-Blue hTLR2 (TA-Lpp and DA-Lpp responsive), HEK-Blue hTLR2-TLR1 (TA-Lpp responsive), and HEK-Blue hTLR2-TLR6 (DA-Lpp responsive) cells cultured and assayed as described above. Serial dilutions of heat-killed bacterial extracts were prepared as described above except bacterial cultures were grown to mid-log-growth phase (optical density at 600 nm [OD_600_] of 1.0 to 1.5) with aeration in 14-ml culture tubes to limit accumulation of DA-Lpp during stationary phase (92). CFU/ml were obtained by plating three different dilutions of cultures on TSA and enumerating colonies after overnight incubation.

### Total RNA Northern blotting.

Northern blots were performed using a NorthernMax kit (Ambion) according to the manufacturer’s instructions. Briefly, 1.5 μg of total RNA for each strain of S. aureus were separated on a 1% MOPS (morpholinepropanesulfonic acid)-formaldehyde-agarose gel and transferred to a BrightStar-Plus positively charged nylon membrane (Invitrogen) using a Whatman Nytran SuPerCharge TurboBlotter kit (GE Healthcare Life Sciences) for 3.5 h. Samples were cross-linked to the membrane by baking at 80°C for 20 min. Biotin-labeled RNA probes were synthesized from DNA with gene-T7-specific primer sets (see Table S1 in the supplemental material) using a MaxiScript T7 transcription kit (Thermo Fisher), including the optional DNase digestion and cleanup with NucAway spin columns (Invitrogen). Probes were added to 10 ng/ml in Ultrahyb ultrasensitive hybridization buffer (Invitrogen), followed by incubation at 72°C for 16 h. The membranes were washed as directed using a NorthernMax kit, with the two high-stringency washes performed at 68°C. RNA was visualized with a chemiluminescent nucleic acid detection kit (Thermo Fisher) according to the manufacturer’s instructions.

### Immunoblotting for strep-tagged Lpp probe.

A plasmid expressing a 10-amino-acid fragment of the SitC Lpp with a C-terminal strep epitope under the control of the strong constitutive promoter *P*_tuf_ was constructed in the shuttle vector pLI50. The plasmid pLI50-sitC10AA was transformed into various RN4220 strains, and cultures were grown to early log phase (OD_600_ = 0.5). Bacterial pellets were obtained by centrifugation, washed once with PBS, and resuspended in buffer (10 mM Tris-HCl [pH 8.0]) containing 50 μg/ml of lysostaphin. Samples were incubated for 15 min at 37°C before being quenched with 4× SDS-PAGE loading buffer. Samples were then heated at 70°C for 15 min before being clarified by centrifugation (18,000 × *g*, 5 min). Aliquots of supernatant were loaded onto an 18% Tris-tricine minigel and separated by electrophoresis using the Tris-tricine running buffer system (93). Protein was transferred to a nitrocellulose membrane (0.2 μM) and developed with an HRP-anti-strep tag conjugate as instructed by the manufacturer (StrepMAB-Classic HRP conjugate; IBA Life Sciences).

### MALDI-TOF mass spectrometry.

Lpps were prepared for mass spectrometry as previously described (20, 43). Briefly, Lpps were extracted using the Triton X-114 phase partitioning method, separated with a 10% SDS-PAGE gel, and transferred to a nitrocellulose membrane. Bands corresponding to S. aureus SitC (SAOUHSC_00634) and to a periplasmic binding protein type 2 family (SAOUHSC_02699) lpp were trypsinized overnight. After elution from the membranes, samples were mixed with α-cyano-4-hydroxycinnamic acid (α-CHCA) matrix and analyzed on an Ultraflextreme (Bruker Daltonics) MALDI-TOF mass spectrometer in positive reflector mode. MS-MS spectra were acquired in Lift mode.

### Cytokine release assay.

The cultivation of HEK-Blue hTLR2 cells and bacterial preparation for the stimulation assay were performed as described previously (23). Cells were cultured in DMEM (Thermo Fisher) supplemented with 10% fetal bovine serum, 50 mg/liter Normocin (InvivoGen), and 1× HEK-Blue Selection (InvivoGen) at 37°C with 5% CO_2_ supplementation. HEK-Blue hTLR2 cells were seeded with 5 × 10^4^ cells/200 μl/well into 96-well cell culture plates, followed by incubation at 37°C with 5% CO_2_ for 24 h. Bacterial cells from overnight culture with antibiotics added according to plasmids being carried (Fig. S8) were harvested and washed three times with Dulbecco PBS (DPBS) before measuring the OD_578_ in DPBS. To calculate bacterial dosage (MOI [multiplicity of infection]), bacteria were set to OD_578_ of 1.0, which equals 1 × 10^8^ CFU/ml. The final bacterial dosage (MOI of 2) was suspended in 50 μl of the HEK-Blue hTLR2 medium and added to the cultured HEK-Blue hTLR2 cells (total volume of medium, 200 μl). Stimulation by these bacteria was carried out for 18 h before cellular supernatants were collected for cytokine assays. IL-8 secreted was measured by using an IL-8 human ELISA kit (Thermo Fisher) according to the manufacturer´s instruction.

10.1128/mBio.01619-20.9 FIG S8 Plasmid maps of pCX-LnsA/LnsB, pCX-LnsA, and pCX-LnsB used in complementation studies in S. aureus USA300 strain JE2. Download FIG S8, TIF file, 0.3 MB. Copyright © 2020 Gardiner et al. 2020 Gardiner et al. This content is distributed under the terms of the Creative Commons Attribution 4.0 International license.
