# Supplementary figures and images for: Lipoprotein N-Acylation in Staphylococcus aureus Is Catalyzed by a Two-Component Acyl Transferase System
Source: mBio. 2020 Jul 28;11(4):e01619-20. doi: 10.1128/mBio.01619-20 (PMC7387801; doi:10.1128/mBio.01619-20)

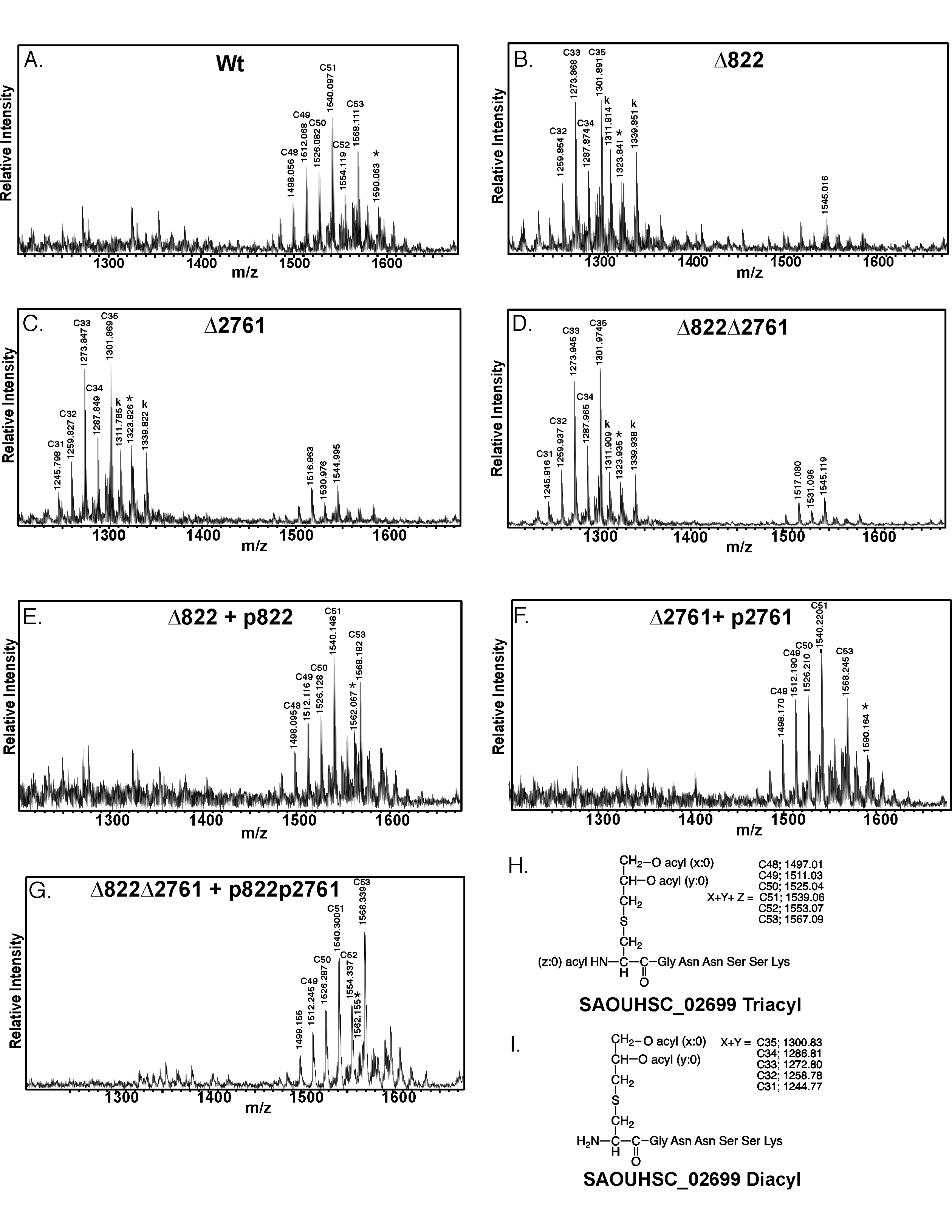

Supplement: FIG S4 [file mBio.01619-20-sf004.tif]
