# Supplementary material for: Lipoprotein N-Acylation in Staphylococcus aureus Is Catalyzed by a Two-Component Acyl Transferase System
Source: mBio. 2020 Jul 28;11(4):e01619-20. doi: 10.1128/mBio.01619-20 (PMC7387801; doi:10.1128/mBio.01619-20)
Supplement: TABLE S1 [file mBio.01619-20-st001.docx]

**Supplemental Materials**

Table S1 **Primers used in this study.**

| Primer Name | Description | Primer Sequence |
| --- | --- | --- |
| JG2031 | Sequencing Primer 3_TaqI ITR1 Fw map Tn insert | cttaagagtgtgttgatagtgcattatc |
| JG2032 | Sequencing Primer 4_TaqI ITR1 Rv map Tn insert | ctttagggttaaccatacgcaagaccaatcac |
| JG2033 | Sequencing Primer 5_TaqI ITR2 Fw map Tn insert | gtaccaaaatccgttcctttttcatagttcc |
| JG2034 | Sequencing Primer 6_TaqI ITR2 Rv map Tn insert | ggtaccataaacggtcgtttatggtaccattc |
| JG2064 | SAOU822 P1 EcoRI deletion vector | gacggccagtgaatttaagcgctatttaaagtg |
| JG2065 | SAOU822 P2 deletion vector | ggatacgacaacgaataatataag |
| JG2066 | SAOU822 P3 deletion vector | ttcgttgtcgtatcctacaaactaaggcttg |
| JG2067 | SAOU822 P4 HindIII deletion vector | tgattacgccaagctaagcgccaatacagaac |
| JG2068 | SAOU822 upcheck deletion allele PCR | ggtgtgttatcattttgaaaacccatg |
| JG2069 | SAOU822 downcheck deletion allele PCR | cttttcatctgtccgacttttgattttg |
| JG2070 | SAOU2761 P1 EcoRI deletion vector | gacggccagtgaattcttttcagcttccatatg |
| JG2071 | SAOU2761 P2 deletion vector | ttcggtagcgtattcttcttctatg |
| JG2072 | SAOU2761 P3 deletion vector | gaatacgctaccgaagcagttatactcgttttg |
| JG2073 | SAOU2761 P4 HindIII deletion vector | tgattacgccaagctcgtaggtatttggaatgc |
| JG2074 | SAOU2761 upcheck deletion allele PCR | caacatttcggattttagcaatttcttcg |
| JG2075 | SAOU2761 downcheck deletion allele PCR | cgattccaaacttaatcggaccattag |
| TM2145 | Bc lit XbaI for Lyso-form lipoprotein synthesis | tattgtcgactctagactttatgatggttagaac |
| TM2146 | Bc lit AscI rev Lyso-form lipoprotein synthesis | tttagaataggcgcgcccgttttacaaagaaaaaaattc |
| KA478 | lgt KO upstream check deletion allele PCR | gatatgaaccatgtaaattaagcaagc |
| KA479 | lgt KO downstream check deletion allele PCR | cgagatataaataacacattgcc |
| TM491 | lsp upstream check deletion allele PCR | cagaggtaaaagagtactacctcg |
| TM492 | lsp downstream check deletion allele PCR | accatactcagtaaaaatgttaacc |
| TM2120 | Pnative SA822 pCN59 for SphI express in *S. aureus* | agctggcggccgctgcatgcctgttttttattcttaatagac |
| TM2121 | SA822 pCN59 rev AscI express in *S. aureus* | attagaataggcgcgccctatttgatgaaactgtg |
| TM2142 | SA2761 Pnative SphI pCN59 express in *S. aureus* | agctggcggccgctgcatgcccccaaaatttcaattc |
| TM2138 | P_pen_ for for pCN59 SphI express in *S. aureus* | gcggccgctgcatgcaagctaattccggtg |
| TM2139 | P_pen_ rev for pCN59 express in *S. aureus* | tctagagtcgacaatatttgattg |
| TM2140 | SA822 for Ppen express in *S. aureus* | attgtcgactctagattcttcaacaaggagc |
| TM2141 | SA2761 for Ppen express in *S. aureus* | attgtcgactctagaatacaactatttaagagg |
| TM89 | pLI50 seq for check for pLI50 plasmids | gctgaaagatcgtacgtacc |
| TM90 | TT seq rev check for terminator on plasmids | caaaattatacatgtcaacg |
| KK1183 | pCN59 seq fwd check for pCN59 plasmids | gtattaccgcctttgagtgagc |
| TM1111 | P_Tuf_ SA for HindIII promoter cloning | aaacctacagaagcttacttacactatgtac |
| TM1112 | P_Tuf_ SA rev promoter cloning | tctagagtcgacaatattataaaatctctcc |
| TM495 | SitC strep tag for XbaI 10 aa SitC fragment | tattgtcgactctagacgaatagaaagaaacg |
| TM1113 | SitC short frag rev BamHI 10 aa SitC fragment | acgctgaaccggatccatcactgctttgtttac |
| TM1817 | pPL2 Ppen for BamHI cloning into pPL2 | tagaactagtggatcaagctaattccggtgg |
| TM1928 | pPL2 TT rev KpnI cloning into pPL2 | agggaacaaaagctgcctgtcactttgcttg |
| KC2134 | SAOUHSC_02761 T7-3’ Northern blot probe | gtttataatacgactcactatagggagagccatttatatttagcac |
| KC2135 | 5’-SAOUHSC_02761 T7 Northern blot probe | gaagaatacgctaccgaacaatcgc |
| KC2136 | SAOUHSC_00822 T7-3’ Northern blot probe | gtttataatacgactcactatagggagatacaccaaatttataagc |
| KC2137 | 5’-SAOUHSC_00822 T7 Northern blot probe | gttacaaaaggccctgtcatgtggg |
| P145 | Reverse primer of pCX-LnsA/LnsB | TGTATTTGACATAGTATACCTCTTA*TTA*ATAAGCTTTAACAAGCCTTAG |
| P146 | Forward primer of pCX- LnsA/LnsB | CTAAGGCTTGTTAAAGCTTAT*TAA*TAAGAGGTATACTATGTCAAATACA |
| P149 | Forward primer of pCX-LnsA | aaccaactaaaatgtaggatcAACAAGGAGCTTTTAAGTGAACTATA |
| P150 | Reverse primer of pCX-LnsA | ataaagcgttcatcattaccc*TTA*ATAAGCTTTAACAAGCCTTAGT |
| P151 | Forward primer of pCX-LnsB | aaccaactaaaatgtaggatcTAAGAGGTATACTATGTCAAATACAA |
| P152 | Reverse primer of pCX- LnsB | ataaagcgttcatcattaccc*TTA*CATGAAAATATGCAAAACGAG |

Lower case letter means sequence of the vector region and capital letter means the chromosomal gene region. Underline indicates start codon and italic indicates stop codon.
